# Interaction between physical exercise and APOE gene polymorphism on cognitive function in older people

**DOI:** 10.1590/1414-431X202010098

**Published:** 2020-12-09

**Authors:** M.E.S. Colovati, I.P. Novais, M. Zampol, G.D. Mendes, M.C.S. Cernach, A. Zanesco

**Affiliations:** 1 Laboratório de Fisiopatologia do Envelhecimento, Programa de Pós-Graduação em Saúde e Meio Ambiente, Universidade Metropolitana de Santos, Santos, SP, Brasil; 2 Departamento de Saúde I, Programa de Pós-Graduação em Educação Física UESB/UESC, Universidade Estadual do Sudoeste da Bahia, Jequié, BA, Brasil

**Keywords:** Alzheimer’s disease, Exercise, Cognition, Apolipoproteins E, Genetic polymorphism, Aged

## Abstract

We aimed to present an overview of the literature regarding the interaction between physical exercise and APOE gene polymorphism on cognitive function, particularly in patients with Alzheimer's disease (AD). Firstly, this review focused on the effect of the physical exercise on cognitive function, regardless of APOE gene polymorphism. Some studies have shown that a high level of cardiorespiratory fitness is associated with less neuronal damage with an improvement in memory score tests whereas other studies failed to detect any association between physical exercise and cognitive improvement either in healthy individuals or patients with AD. Taken together, standardized protocols and more longitudinal studies are required to provide a better insight into the effects of physical exercise on cognitive function. Although there is no agreement in the literature regarding the effects of physical exercise on cognitive function, it is well established that it improves social interaction and the feeling of well-being, thereby positively contributing to the quality of life of the elderly. Regarding the influence of physical exercise on cognitive function in APOE ε4 allele carriers, the data trend shows that the carriers of allele ε4 for APOE gene were more responsive to the beneficial effects of physical exercise on cognitive function compared with non-carriers. Nevertheless, studies with larger sample sizes will provide more accuracy about this relationship.

## Introduction

Advances in biomedical research have resulted in higher life expectancy (1). On the other hand, increased longevity has led to a rise in the prevalence of neurodegenerative diseases, which are associated with high costs in the health care system. In addition, the diagnosis of some of these diseases is very difficult and several causal factors are involved (2). Several types of dementia exist and Alzheimer's disease (AD) is the most prevalent one (approximately 60-80% of the cases) followed by vascular dementia (20-30%) and frontal-temporal dementia (10%) (3). Although many studies have revealed the neurobiology of AD, much is yet to be discovered related to the underlying mechanisms by which this disease affects neural regulation and its function.

Evidence has shown that APOE-ε4 carriers have an increased risk for AD, with heterozygous carriers of one ε4 allele being 3-4 times more likely to develop this neurodegenerative disorder than non-carriers, and with the risk for homozygous carriers being even higher (4).

The practice of physical exercise is considered a useful intervention for preventing the decline in cognitive function or for attenuating the risk factors for AD such as arterial hypertension, dyslipidemias, type II diabetes mellitus, and metabolic syndrome (3). However, the beneficial effect of physical exercise on AD is not clear. Given that pharmacological treatments have been ineffective both in the prevention and attenuation of the progression of AD (5), it is important to examine how a lifestyle intervention such as physical exercise would mitigate the deleterious effects of AD on daily activities in an attempt to improve the quality of life of the elderly. Therefore, this review aimed to address the interaction between physical exercise and the presence of polymorphism in APOE on cognitive function in older people.

In order to provide a clinical basis for AD, we summarized some concepts that are currently accepted, and subsequently, we focused on the current knowledge about the possible effects of physical exercise on cognitive function in APOΕε4 allele carriers.

## Material and Methods

### Search strategy

The methodological design of this study consisted of the search and analysis of articles that verified the effects of physical exercise on cognitive function in APOEε4 allele carriers, particularly in patients with AD. The bibliographic search was conducted on the following databases: Web of Science, Pubmed, Biological Abstracts, PsycINFO, and Scopus, from 2000 to 2020.

The following keywords and Boolean operators were used: physical exercise OR physical activity OR exercise OR training AND cognition OR genetic polymorphism OR apolipoprotein E AND Alzheimer’s disease OR aged OR older OR elderly. Besides the database search, a manual search was carried out on the references from the selected articles. The search was conducted in January of 2020. Afterward, articles were analyzed in the following order: 1) title analysis; 2) abstract analysis; 3) whole article analysis; and 4) selection of articles.

### Inclusion criteria

Inclusion criteria were i) human studies and ii) interventions with physical exercise/physical activity, excluding any diet intervention and/or supplementation.

## Results and Discussion

After a search based on the established criteria, 1,548 articles were found. In the first analysis, 828 articles were excluded as the title was not related to the aim of this study, and 664 articles were excluded by their abstracts. Thus, the reading of the 56 selected articles was performed for this review.

### Pathophysiology of AD

**Figure 1:**
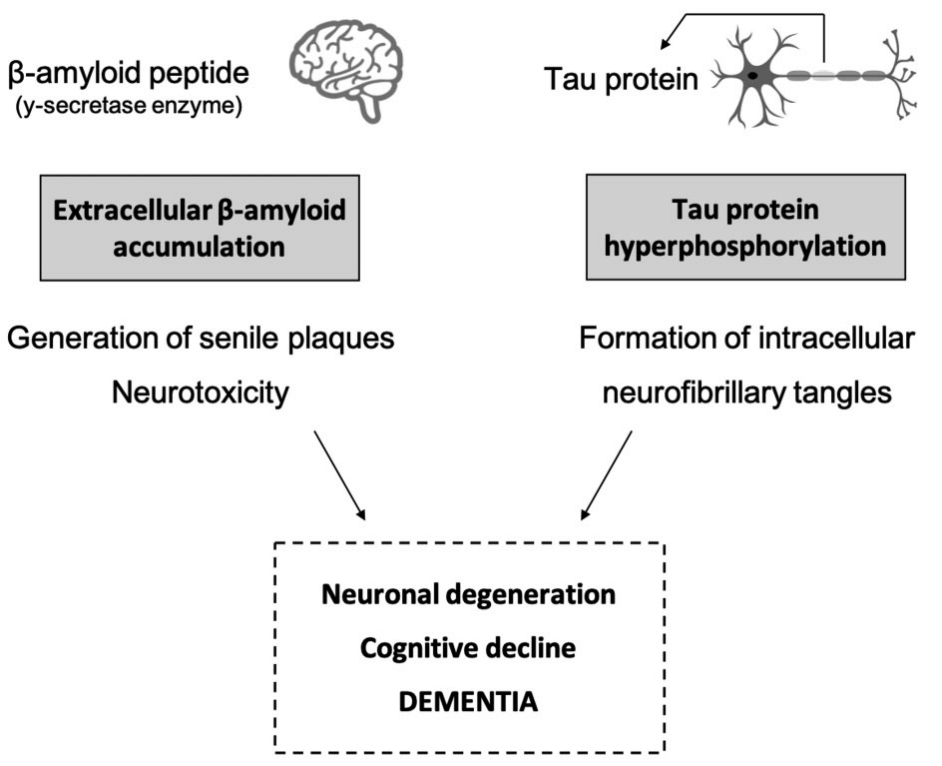
Current hypothesis of the physiopathological mechanisms involved in Alzheimer's disease.

AD, accounting for 60-80% of all cases of dementia, is characterized by progressive loss of cognitive function, changes in behavior, and loss of motor capacity, thereby affecting the patient's quality of life (6,7). The causes of AD are unknown, and its etiology is multifactorial involving genetic, epigenetic, and environmental factors (8,9). The most widely accepted hypothesis for the pathogenesis of AD is that the accumulation of β-amyloid peptide (βA) in the brain leads to neurotoxicity. βA is derived from the amyloid precursor protein (APP) via two pathways - one involving the enzyme α-secretase that produces SAPPα, which plays a neuroprotective role, and another involving γ-secretase that produces βA - and its degradation is catalyzed by the enzyme neprilysin. Under physiological conditions, the balance between βA production and degradation is maintained by the action of this enzyme and an imbalance leads to its accumulation and a decline in cognitive function and neuronal death (10). Accumulation of hyperphosphorylated neurofibrillary tangles of tau proteins was observed parallel to the accumulation of βA. Tau, a protein found in the axons, binds to microtubules and promotes their assembly and stability, and its phosphorylation is regulated by the balanced action of multiple enzymes. In AD, hyperphosphorylation of tau results in the formation of neurofibrillary tangles that compromise axonal transport and neuronal function. Accumulation of extracellular βA and tau hyperphosphorylation in the neuron result in progressive cerebral atrophy, with neuronal degeneration and dementia (11). Please see Figure 1 for more details.

AD can be classified as familial (1-5% of cases) or sporadic (95% of cases) (7). Inherited dominant mutations in the APP and neprilysin genes are implicated in the pathophysiology of early-onset AD (12). Consequently, these genes are considered biomarkers for the disease. On the other hand, the etiology of the sporadic form, which shows a late onset (after 65 years), has not yet been elucidated (2,13). Therefore, the analysis of polymorphisms in candidate genes has contributed to risk assessment and the primary prevention of several chronic degenerative diseases, including diabetes mellitus, myocardial infarction (14), and AD (15).

### Apolipoprotein E (APOE)

APOE plays a regulatory role in the central nervous system (CNS), acting in the distribution and transport of cholesterol to the nerve tissues for the maintenance of their integrity (neuroplasticity) as well as in the immune response and mitochondrial function of neurons (16). APOE, one of the main CNS lipoproteins, is synthesized peripherally in the liver and in the CNS by the astrocytes and has three isoforms - APOEε2, APOEε3, and APOEε4 - with two important functional domains: the N-terminal domain that contains its receptor binding sites and the C-terminal domain that binds to βA14 (17). APOE is located in chromosome 19q13.2 and codes for the three common alleles (ε2, ε3, ε4). Carriers of the homozygous or heterozygous ε4 allele have increased genetic risk for late onset AD (17). A recent study of neuroimaging and neuropathology has shown that APOEε4 carriers have abundant and accelerated βA deposition compared with non-carriers (18).

### AD and physical exercise

The beneficial effects in both experimental models and humans of physical exercise on hypertension, type 2 diabetes mellitus, and dyslipidemia, which are considered risk factors for AD, are well established (19–21). However, some studies have failed to clearly show whether physical exercise prevents AD or mitigates its progression, either due to methodological difficulties or differences in physical exercise protocols (22,23). It is believed that the beneficial effects of physical exercise on cognitive function are indirect (24). A classic example is the fact that individuals who experienced ischemic events are more likely to develop AD, with a higher production of reactive oxygen species resulting in the accumulation of βA, and physical exercise was shown to act as an antioxidant and to improve some types of AD (25,26 [Bibr B27]).

Studies that have shown the beneficial effect of physical exercise on cognitive function, as well as those in which no changes were detected, are described below, and we subsequently address the interaction between physical exercise, APOE polymorphism, and cognitive function.

### Beneficial effects of physical exercise

Studies have shown that physical exercise has systemic positive effects on AD via the induction of angiogenesis and anti-inflammatory and antioxidant activities (26–28), whereas other studies have shown that physical exercise promotes neurogenesis as well as reduces tau protein hyperphosphorylation and excessive Aβ production (29). However, several studies have been conducted with animal models and it is known that experimental models of AD do not reflect the changes observed in humans. Considering this context, we present details of some clinical studies.

A previous study including healthy men showed that exercise training for approximately 2 years without direct supervision improved cerebral blood flow compared with physically inactive individuals (30). Another study evaluated elderly individuals with normal cognitive function, mild dysfunction, or AD. The practice of physical exercise was associated with higher brain volume in all groups and inversely correlated with body mass index (31). A similar study showed a positive association between cardiorespiratory fitness and white brain mass integrity in elderly individuals (32). Recently, it was observed that elderly individuals with mild cognitive dysfunction who practiced physical exercise exhibited improvement in the memory score that was associated with reduced atrophy of the hippocampus (33). Consistent with these findings, an inverse relationship was observed between βA42 concentration and cognitive dysfunction in individuals with high cardiorespiratory fitness (34,35).

Studies involving the direct application of an exercise training program or direct supervision of physical activity in the elderly are scarce and the greatest limitation is adherence. A study to evaluate patients with AD undergoing 12 weeks of physical activity showed a positive association between aerobic capacity and cognitive test scores (36). Another study showed improvement in cognitive test scores of patients with AD and in the peak value of VO_2_ (37). Further, a recent review showed that improvement in cardiorespiratory fitness promotes the release of systemic mediators that potentially act on the CNS, thereby preventing the neuronal damage of AD (38). Table 1 summarizes these studies.

**Table 1:** Studies showing beneficial effects of physical exercise on cognitive function.

Reference	Participants	Methodology	Type of study	Main findings
Ainslie et al. (2008) 30	Healthy men PA >2 years (n=153) PI >2 years (n=154)	Cerebral blood flow by Doppler VO_2_max	Cross-sectional	Improvement of the cerebral blood flow in PA compared with PI individuals
Boyle et al. (2015) 31	963 participants >65 years. 10% with AD, MCI, control	MRI scan BMI measurements Level of physical activity	Longitudinal	High level of PA was associated with higher brain volume Higher BMI was inversely correlated with cerebral volume
Perea et al. (2016) 32	Individuals with AD >55 years PA (n=40) PI (n=37)	26 weeks of physical exercise MRI scan VO_2_max	Longitudinal	Higher level of CRF was associated with white brain mass integrity
Morris et al. (2017) 33	Individuals >65 years PA (n=34) PI (n=34)	MRI scan VO_2_max	Longitudinal	Improvement in CRF was associated with better cognitive test score and reduction of hippocampus atrophy
Schultz et al. (2015) 34	Individuals >65 years (n=69)68% women 72% family history of AD	βA42 level in the CSF PET scan VO_2_max	Cross-sectional	Positive relationship between VO_2_ peak and βA42 levels
Law et al. (2018) 35	Individuals >55 years (n=85) 61% women 81% family history of AD	Accelerometer for 7 days Levels of βA42 and tau in CSF	Longitudinal	Physical activity was associated with increment in βA42 and lower rate of p-tau/βA42
Holthoff et al. (2015) 36	Patients with AD >65 years 50% women Exercise training (n=15) Control (n=15)	12 weeks of exercise training Cognitive and motor tests	Longitudinal	Improvement of cognitive and motor functions in trained group
Sobol et al. (2018) 37	Patients with MCI: 50-90 years Physical exercise (n=26) Control (n=23)	16 weeks of exercise training Cognitive test VO_2_max	Longitudinal	Improvement in VO_2_max was associated with better score of cognitive tests in trained group
Seifert et al. (2010) 39	Young men Physical exercise (n=7) Control (n=5)	Three months of exercise training Plasma BDNFVO_2_max	Longitudinal	Higher VO_2_max was associated with increment in BDNF levels in trained group

PA: physically active; PI: physically inactive; VO_2_max: maximum oxygen consumption; AD: Alzheimer's disease; MCI: mild cognitive impairment; MRI scan: magnetic resonance image; BMI: body mass index; CSF: cerebrospinal fluid; βA: peptide β-amyloid; CRF: cardiorespiratory fitness; PET scan: positron emission tomography; HR: heart rate; BDNF: brain-derived neurotrophic factor.

Moreover, neurotrophic factors (NTFs) have been evaluated in the response to physical exercise because they play an important role in memory formation, cognitive capacity, and neuron survival, and physical exercise improves neuroplasticity. The most studied NTFs are neural growth factor, brain-derived neurotrophic factor (BDNF), and neurotrophins (26,39,40). However, the mechanisms by which physical exercise/training would promote these effects in AD remain unclear.

### Absence of beneficial effects of physical exercise

Contrary to the above-mentioned studies, a systematic review showed that a conclusion about beneficial effects of physical exercise cannot be drawn from the studies conducted. However, a multi-domain intervention that associates physical activity, diet, and cognitive training was shown to improve cognitive abilities among the elderly (41). With regard to cerebral blood flow, exercise training did not modify the Mini-Mental State Exam scores and did not lead to changes in cerebral blood flow (42). In fact, a recent study evaluating brain damage biomarkers or inflammatory mediators in cerebrospinal fluid in patients with AD who trained found no changes after the intervention (43). A recent systematic review examining the relationship between physical activity and several biomarkers for AD concluded that the quality of the studies does not facilitate a definitive conclusion and that most of the studies analyzed showed no association between physical exercise and cognitive improvement (44).

Therefore, standardized protocols and larger and more detailed randomized controlled clinical trials with long-term follow-up are required to provide a better insight into the effects of physical exercise on cognitive function (45–47).

It should also be emphasized that the beneficial effects in the elderly have been observed more consistently in participants without dementia, whereas the results are controversial in patients with AD, suggesting that the practice of physical exercise has preventive effects on brain activity and that the potential existence of various forms of AD generates disparate results (48). Although there is no agreement in the literature regarding the effects of physical exercise on cognitive function, it is well established that it improves social interaction and the feeling of well-being, thereby positively contributing to the quality of life of the elderly. Therefore, public policies to encourage the practice of physical exercise are fundamental to minimize the prevalence of AD or delay its onset. Table 2 summarizes these studies.

**Table 2:** Studies showing absence of beneficial effects of physical exercise on cognitive function.

Reference	Participants	Methodology	Type of study	Main findings
Brasure et al. (2018) 41	16 articles (2009-2017)	Systematic review Patients with AD *vs* control underwent physical activity for 6 months	Longitudinal	Failed to find an association between physical activity and cognitive function
van der Kleij et al. (2018) 42	Patient with mild or moderate AD >65 years 40% women Exercise training (n=27) Control (n=24)	16-week exercise training, 60min, 3x-week, 70-80% HRR MRI scan VO_2_max	Longitudinal	Greater VO_2_max in trained group,no change in cerebral blood flow between groups or MEEM score
Jensen et al. (2017) 43	Patients with AD >65 years 48% women Exercise training (n=25) Control (n=26)	16-week exercise training CSF for measurements of biomarkers	Longitudinal	Exercise training did not promote any changes in the brain damage biomarkers or neuroinflammation
Frederiksen et al. (2019) 44	Jan/1984 to Feb/2018 34 studies/healthy participants 3 studies/healthy/MCI participants 1 study/MCI participants 2 studies/AD patients	Systematic review Biomarkers, MRI scan, PET	Longitudinal	No association between physical exercise and cognitive function
Vidoni et al. (2013) 48	Individuals 60-85 years ESAD (n=16) Control (n=18)	MRI scan VO_2_max Motor tests	Cross-sectional	Cardiorespiratory fitness was associated with improvement in brain activity in control group but not in patients with ESAD

AD: Alzheimer's disease; HRR: heart rate reserve; MRI scan: magnetic resonance image; VO_2_max: maximum oxygen consumption; CSF: cerebrospinal fluid; MEEM: mini-mental state exam; PET: positron emission tomography; MCI: mild cognitive impairment; ESAD: early stage of AD.

### Interaction between APOE genetics and physical exercise on cognitive function

A previous study showed that physically active individuals who were APOEε4 allele carriers exhibited a stable course of cognitive performance and protection against hippocampal atrophy during an 18-month follow-up period compared with physically inactive alleloε4 carriers. This suggests that physical exercise offers protection against AD neurodegeneration even in carriers of the APOΕε4 allele (49). In a recent study evaluating participants aged ≥65 years (362 carriers of the APOΕε4 allele and 747 non-carriers), it was concluded that multi-domain interventions (diet, physical exercise, cognitive training, and vascular risk monitoring) were beneficial for cognitive function, even in patients with the APOΕε4 allele. However, patient adherence remains a challenge (50). Another study evaluating 1,438 elderly individuals for 6.5 years showed that physical exercise was able to mitigate dementia risk in participants with the APOΕε4 allele compared with physically inactive elderly individuals (51). The association between cardiovascular risk, educational level, and leisure activity has also been studied in patients with the APOEε4 allele and the findings show that those with low vascular risk, high educational level, and who practiced leisure activities have a low dementia risk (52). A recent study showed that APOEε4 allele carriers are more responsive to exercise training, with better performance in motor and cognitive tests compared with non-carriers of the allele (53).

In contrast, other studies have failed to detect any effect of physical exercise on dementia in patients who carry ε4 allele. One study showed that the likelihood of developing dementia is not significantly different between those who exercise and those who do not (54). In agreement with that, it has been shown that exercise training did not modify the cognitive function in patients with the APOEε4 allele (55). Moreover, the APOEε4 allele carriers have a lower plasma concentration of BDNF, and exercise training does not promote changes in the levels of this neurotrophic factor (56). Table 3 summarizes these studies.

**Table 3:** Studies showing interaction between APOE gene polymorphism, physical exercise, and cognitive function.

Reference	Participants	Methodology	Type of study	Main findings
Smith et al. (2016) 49	Healthy individuals >65 years (n=88)	Level of PA Neuroimage Cognitive test Genotype for APOE	Longitudinal	High PA level has protective effect on neurodegeneration in patients who carry APOEε4 allele
Solomon et al. (2018) 50	Individuals >65 years APOEε4 carriers (n=362) APOEε4 non-carriers (n=747)	Multi-domain tasks Cognitive test Genotypes for APOE	Longitudinal	APOEε4 carriers or non-carriers are responsive to multi-domain tasks
Shih et al. (2018) 51	Individuals >60 years (n=1,438)	Level of PA Cognitive test Genotype for APOE	Longitudinal	Higher PA level mitigates dementia in APOEε4 allele carriers
Ferrari et al. (2013) 52	Individuals >75 years (n=932)	Educational level Leisure activities Genotypes for APOE	Longitudinal	Higher educational, lower vascular risk, and leisure activities diminish risk of dementia in patients who carry APOEε4 allele
Jensen et al. (2019) 53	Patients with AD >65 years APOEε4 carrier (n=17) APOEε4 non-carrier (n=5)	16 weeks of AET, 60 min, 3x/week, 70-80% HR reserve Cognitive and motor tests Genotype for APOE	Longitudinal	APOEε4 allele carriers are more responsive to AET than non-carriers
Fenesi et al. (2017) 54	Healthy individuals >65 years (n=1,646)	PA levelGenotype for APOE	Longitudinal	Physical exercise had no effect on dementia in patients who carry APOEε4 allele
Stern et al. (2019) 55	Individuals 20-67 years (n=132)	AET Cognitive test Genotype for APOE	Longitudinal	AET does not modify the score of cognitive tests in APOEε4 carriers
Allard et al. (2017) 56	Individuals with MCI >55 years (n=21)	AET for 6 months Genotype for APOEBDNF level	Longitudinal	No changes in BDNF level after AET in APOEε4 carriers

PA: physical activity; AD: Alzheimer's disease; BDNF: brain-derived neurotrophic factor; HR: heart rate; AET: aerobic exercise training; MCI: mild cognitive impairment.

### Conclusion

In general, physical exercise might be effective in ameliorating the score of cognitive tests and likely prevent neurodegeneration in individuals who have genetic risk factors, such as APOEε4 allele carriers. Nevertheless, studies with larger sample sizes will provide more accuracy in this relationship.
